# Rescuing impacted biliary extraction basket using cholangioscopy-directed lithotripsy and a pulley system

**DOI:** 10.1016/j.vgie.2025.09.008

**Published:** 2025-10-09

**Authors:** Hyun Jae Kim, Rajit Gilhotra, Andrew Fetz, Eric C.S. Lam

**Affiliations:** 1 University of British Columbia, Department of Medicine, Vancouver, British Columbia, Canada; 2 Royal Brisbane and Women's Hospital, Department of Gastroenterology, Brisbane, Australia

## Abstract

**Background and Aims:**

Impaction of a biliary extraction basket during endoscopic retrograde cholangiopancreatography (ERCP) is a rare but challenging adverse event. We describe a novel endoscopic rescue approach using cholangioscopy-guided lithotripsy and an improvised pulley system to remove an impacted biliary basket after failed transoral mechanical lithotripsy.

**Methods:**

A 52-year-old man presented with obstructive jaundice because of a 9-mm common bile duct stone. Initial ERCP with mechanical lithotripsy using an extraction basket resulted in basket impaction. Rescue lithotripsy with a transoral lithotriptor resulted in wires fracturing at the handle. Rescue endoscopic management of the impacted basket was performed at our center. The patient had significant duodenal ulceration due to tension from the exposed basket wires.

**Results:**

Cholangioscopy-directed electrohydraulic lithotripsy was performed to fracture the entrapped stone. An improvised pulley mechanism using grasping forceps as a fulcrum was used to successfully extract the entrapped basket. The patient was discharged within 48 hours without requiring surgical interventions.

**Conclusions:**

Cholangioscopy-guided lithotripsy is effective for releasing impacted baskets from entrapped stones. A pulley technique using grasping forceps can enable safe basket extraction by altering the vector of wire traction. This strategy provides a minimally invasive solution for managing an impacted biliary extraction basket.

## Introduction

An impacted extraction basket during endoscopic retrograde cholangiopancreatography (ERCP) is an uncommon but challenging adverse event that poses a significant challenge for endoscopists.^1^

## Case report

**Figure 1:**
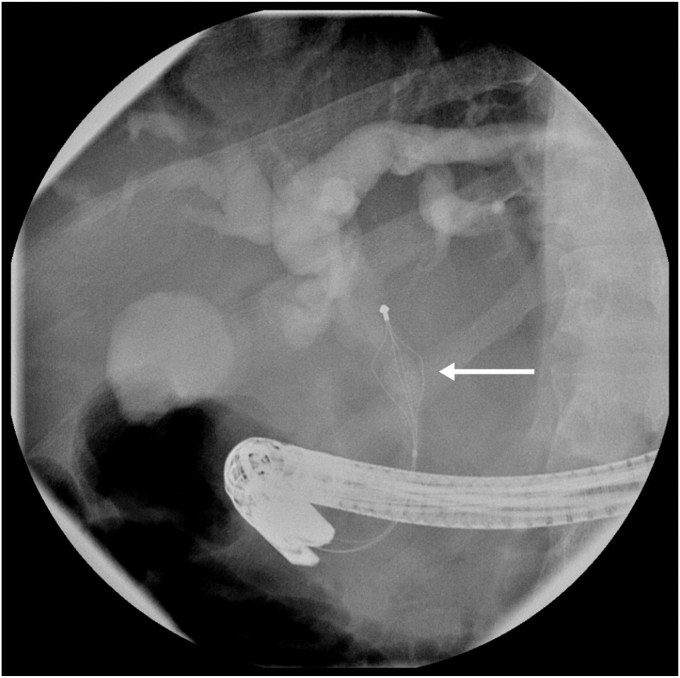
Fluoroscopy showing impacted stone extraction basket with stone in the mid–common bile duct (*arrow*).

A 52-year-old man presented with abdominal pain and jaundice. Ultrasonography demonstrated a 9-mm common bile duct stone. ERCP with sphincterotomy and basket trawl was performed but failed to extract the stone. Mechanical lithotripsy was attempted using a 15-mm trapezoid lithotripsy basket, but the basket wires fractured at the handle outside the mouth, leaving the basket impacted within the bile duct (Video 1, available online at www.videogie.org). Despite 15-mm balloon sphincteroplasty, the basket remained impacted (Fig. 1). Rescue transoral lithotripsy (Soehendra lithotriptor; Cook Medical, Bloomington, Ind, USA) resulted in the wires fracturing at the lithotriptor handle. With less than 10 inches of wire remaining, additional lithotripsy attempt was not feasible. The patient was transferred to our tertiary center with basket wires securely taped to the side of his mouth. The patient was tolerating the exposed wires and secretions; therefore, he was transferred without intubation, accompanied by medical staff prepared for airway support if required.

**Figure 2:**
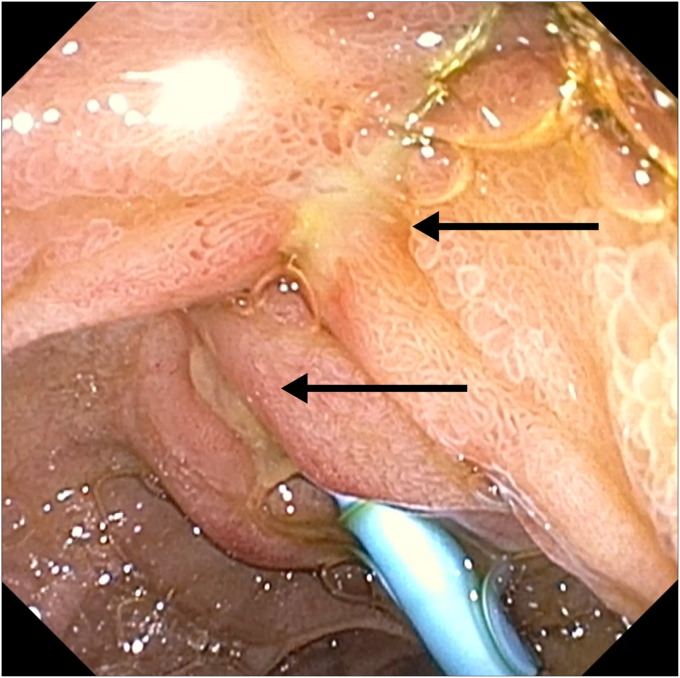
Long linear pressure ulcerations (*arrows*) extending from the ampulla to the esophagus secondary to wire tension from biliary stone extraction basket wires.

**Figure 3:**
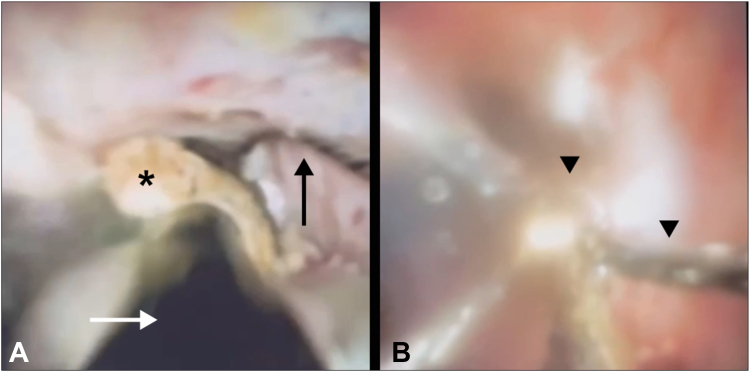
**A,** Basket wires (*black arrow*) with a captured stone (*asterisk*) are visualized during cholangioscopy (*white arrow* indicating electrohydraulic lithotripsy probe). **B,** After cholangioscopy-guided electrohydraulic lithotripsy, the intact extraction basket (*arrowheads*) is seen free of stone debris.

Multidisciplinary preplanning included anesthesia coordination for expedited reintervention and surgical backup if endoscopic rescue failed. Repeat ERCP was performed within 10 hours of the index ERCP with the patient under general anesthesia. A long linear ulcer along the path of the basket wire was seen from the ampulla to the esophagus because of the wire tension (Fig. 2). A large balloon dilation was aborted because of a developing laceration at the apex of the sphincterotomy site along the wire-induced ulceration. Cholangioscopy confirmed a hard stone entrapped within the basket. Electrohydraulic lithotripsy was performed using the medium-high power setting, successfully fragmenting the stone and freeing the basket (Fig. 3).

**Figure 4:**
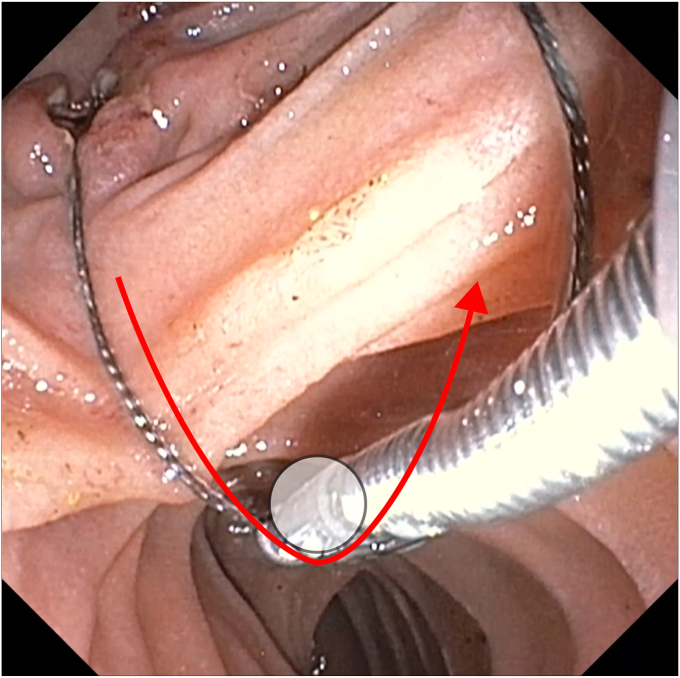
A pulley system improvised to extract the stone basket safely using the grasping forceps as a fulcrum (*white circle*). With use of the grasping forceps as a fulcrum, pulling on the exposed basket wires from the patient's mouth side, resulting in pulling force on the wire toward the distal duodenum away from the linear ulcerations (*red arrow*).

**Figure 5:**
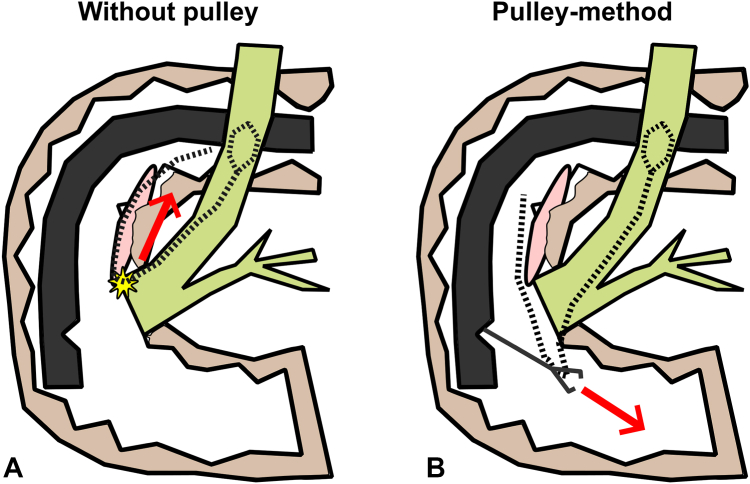
A graphical depiction of the pulley system devised. Without use of the pulley method (**A**), pulling on the exposed basket wires from the patient's mouth would result in tension pressure (*red arrow*) on the tension-induced wire, risking tear or perforation (*star*). With use of the pulley mechanism (**B**), direction of the force can be safely redirected away from the ulcerations (*red arrow*).

Direct traction on wires from the mouth risked worsening the duodenal ulceration and possible perforation. We attempted to pull the lithotripsy basket using grasping forceps, but the wires were too slippery and could not be grasped firmly. To minimize shear on the duodenal wall while extracting the basket, we devised a pulley system using the grasping forceps as a fulcrum to redirect the pulling vector away from the ampulla (Figs. 4 and 5). Simultaneous oral traction and pushing the grasping forceps into the distal duodenum enabled safe extraction of the basket. The patient was observed for 48 hours and then discharged without any adverse events. He later underwent elective cholecystectomy after 8 weeks.

## Discussion

Although modern extraction baskets include built-in safety features to reduce risk of basket impaction, basket impaction can still occur, and endoscopists must be aware of this adverse event and know of appropriate management options. Rescue options include rescue lithotripsy with a transoral mechanical lithotriptor, large balloon dilation, cholangioscopy-guided lithotripsy, or surgery.^2^ A major drawback of transoral lithotripsy is the need to remove the scope and cut the wires, which may necessitate surgery if it fails. Here, we demonstrate a safe and effective endoscopic rescue to an impacted basket after failed transoral mechanical lithotripsy.

Two critical aspects of managing an impacted basket after failed transoral lithotripsy are (1) releasing the basket from the entrapped stone and (2) ensuring atraumatic basket extraction. Cholangioscopy-guided lithotripsy enables safe and effective fragmentation of an entrapped stone without additional dilation or extended sphincterotomy. Atraumatic extraction is essential once the basket is free. Especially in delayed management, exposed basket wires can cause ulceration, and traction from the mouth on the basket may risk additional mucosal injury.^3^^,^^4^ Although cutting the wires in the duodenum using argon plasma coagulation has been described, it carries risks of duodenal injury from thermal injury or exposed wire tips.^5^ As demonstrated in our case, using grasping forceps as a fulcrum to create a pulley system can redirect the pulling force vector and allow for safe basket extraction without additional tools or increased risk.

## Patient consent

Written informed consent was obtained from the patient for publication of this case report and accompanying images.

## Disclosure

The following author disclosed financial relationships: E. C. S. Lam: Speaker for Boston Scientific and Pharmascience. All other authors disclosed no financial relationships.

## Supplementary data

Video 1 demonstrating endoscopic retrieval of an impacted biliary extraction basket using cholangioscopy-guided lithotripsy and an improvised pulley system during ERCP.
